# Growth management and prevalence of underweight of indigenous children (*Orang Asli*) in Peninsular Malaysia: a clinical audit

**DOI:** 10.1186/s12887-022-03532-7

**Published:** 2022-08-10

**Authors:** Chii-Chii CHEW, Hasni-Adha IBRAHIM, Venugopalan K. BALAN, Nor-Azizah ABD-AZIZ, Hooi-Meng PUAH, Amar-Singh HSS

**Affiliations:** 1 Clinical Research Centre Perak, Hospital Raja Permaisuri Bainun, Ministry of Health, Level 4, Ambulatory Care Centre (ACC), Jalan Raja Ashman Shah, 30450 Ipoh, Perak Malaysia; 2 Paediatric Department, Hospital Raja Permaisuri Bainun, Ministry of Health, Jalan Raja Ashman Shah, 30450 Ipoh, Perak Malaysia; 3 Perak State Health Department, Ministry of Health, 30000, Ipoh, Perak Malaysia

**Keywords:** Indigenous population, Growth, Malnutrition, Clinical audit, Growth charts, Child, Child nutrition disorders

## Abstract

**Background:**

Most indigenous people (*Orang Asli* in Peninsular Malaysia) live in poverty, and their children are at risk of growth problems due to nutrition deficiency. Routine health and growth assessments are essential to identify these children. This clinical audit aimed to determine the growth management of indigenous children and the prevalence of underweight among these children in Perak state, Malaysia.

**Methods:**

A clinical audit was conducted in 2016 after obtaining consensus from stakeholders for audit criteria, forms, and procedures. All weight-for-age growth charts of *Orang Asli* children aged 2 and below were sampled for retrospective audit. This audit excluded children who required special needs. Growth charts were examined against audit criteria: (i) quality of growth chart plotting (charts were not plotted, incompletely plotted, or incorrectly plotted), (ii) presence of underweight, and (iii) appropriateness of action taken (appropriate or inappropriate action) according to local standard operating policies. Eligible auditors were first trained using simulated growth charts.

**Results:**

Out of 1329 growth charts audited, 797 (60%) growth charts were correctly plotted, 527 (39.7%) were incompletely or incorrectly plotted, and five (0.3%) were not plotted. Overall, 40.0% of the growth chart was plotted incorrectly or completely not plotted. 550 (41.4%) children were found to be underweight, and 71.5% of them received inappropriate care management. Where growth charts were correctly plotted, 283 children were identified with underweight problems, and 194 (68.6%) of them received inappropriate care. For growth charts that were plotted incompletely or incorrectly, 267 children were identified as having underweight problems, and 199 (74.5%) received inappropriate care. The growth status of 265 (19.9%) children was unable to be determined due to incomplete plotting.

**Conclusion:**

Approximately 40% of indigenous *Orang Asli* children aged 2 years and under were underweight, and most of them received inappropriate care.

## Introduction

Underweight (i.e., weight-for-age less than –2SD) is one of the most common growth problems among children in disadvantaged populations, and it is usually attributed to a lack of nutrition, repeated infections, and poverty [1]. Nutrition deficiencies are prevalent in developing countries among children aged 6 months to 2 years, mainly due to early weaning, delayed introduction of complementary foods, poor diet, and severe or frequent infections [2]. In 2015, the WHO estimated that nearly 170 million children across the world were underweight, and that the prevalence was predicted to decline to 113.4 million from 1990 to 2015. A similar reduction trend was predicted in developing countries, where a reduction of 30.2 to 19.3% of underweight children was estimated [3]. Despite this improvement, large segments of the marginalised populations in developing countries still have malnutrition and poverty-related diseases [4].

One of the most marginalised populations in Peninsular Malaysia is the indigenous people, also known collectively as the *Orang Asli*. The majority live below the poverty line with a mean household income of below RM 194 (approximately USD 58.02) per month [5]. This has long been associated with the problem of childhood malnutrition [6]. The prevalence of underweight among *Orang Asli* children under 5 years of age ranged from 32.7 to 65% [7–12], with an even higher prevalence of underweight (78 to 95%) being noted in the *Temuan and Mahmeri* tribes reported by Shasikala, S et al. [13]. The proportions of children who were severely underweight (Z-scores < − 3 SD) and moderately underweight (−3SDZ < scores < −2SD) were 18.4 and 30.3%, respectively [7].

The *Orang Asli* population in Malaysia is distributed in the states of Pahang (67,506; 37.9%), followed by Perak (53,299; 29.9%), Selangor (17,587; 9.9%), Kelantan (13,457; 7.6%), Johor (13,139; 7.4%) and Negeri Sembilan (10,531, 5.9%) [14]. Given the substantial proportion of the *Orang Asli* population in Perak and the lack of research on the growth status of *Orang Asli* children in this state (most such studies were conducted in Pahang state [7, 9, 12, 15–17]), the prevalence of underweight in this state needs to be investigated.

The World Health Organization’s (WHO) Child Growth Standards, also known as growth charts, are an effective paediatric toolkit for characterising the physiological growth of children from birth to 5 years old. A child with normal growth will follow the trend parallel to the median and z-score on the growth chart. If a child has a real or potential growth problem, the growth line will either cross the z-score line, show a sharp incline or decline, or remain flat (stagnant) [18, 19]. Early detection of growth problems is important to ensure appropriate referral, treatment, and intervention can be introduced early to reduce morbidity and mortality in children [20]. Growth charts can only serve their function of monitoring children’s growth when health care providers are able to conduct anthropometric measurements and plot them on the appropriate growth charts correctly [19]. Therefore, apart from determining the prevalence of underweight, the ability of healthcare providers to plot growth charts is crucial and has to be determined.

In addition, studies suggest that the ability to access healthcare services and resources in sustaining the health of a community should not be shouldered by the recipient (or patients), but also cover the dimensions of the providers who deliver health services to the recipient [21, 22]. Therefore, the healthcare providers entrusted with the responsibility of *Orang Asli* children’s growth management have to be assessed. This study aims to evaluate growth management and the prevalence of underweight among indigenous (*Orang Asli*) children under 2 years of age in the Perak state of Malaysia.

## Method

### Study setting

This cross-sectional study was conducted from June to November 2016 at the primary care and mobile health clinics located in 7 out of 10 districts in Perak, Malaysia. These clinics consist of Primary Health Clinics (*Klinik Kesihatan*), Rural Clinics (*Klinik Desa*), Mother-and-Child Health Clinics (*Klinik Kesihaan Ibu dan Anak*), and *Orang Asli* Mobile Clinics (*Klinik Bergerak Orang Asli*) which are frequently visited by the *Orang Asli community* for primary care, dental care, maternal and child’s health care [23].

### Standard care of growth assessment

The maternal and child health monitoring programmes have been started since the 1950’s in all health clinics distributed across Malaysia, and this service has been expanded progressively to meet the current needs [23]. Children aged 0 to 6 years old have access to free child health care services in all government-funded health clinics in Malaysia. All newborns and children, that attend the clinics, undergo continuous growth assessment monitoring and health screening. Their details are recorded in the registry at government-funded health clinics nearest to them. The children’s caregivers (usually the parents) are given a ‘Parent Child Health Record Book’ that also contains growth and monitoring details [24].

At each appointment visit to the clinic, the attendance of the children is recorded in the clinic registry. The ‘Parent Child Health Record Book’ is obtained from the caregivers, and the ‘Clinic Child Health Record’ kept in the clinic is traced. The children’s anthropometric measurements were conducted and charted on the intended growth charts (weight-for-age, length/height-for-age, weight-for-length, and BMI-for-age). The measurements are recorded in the ‘Parent Child Health Record Book’ as well as ‘Clinic Child Health Record’ in the clinic. The growth status is evaluated, determined, and documented. Caregivers are interviewed by the healthcare providers (HCPs) for the children’s nutrition intake, immunisation status, growth and development, and current health and social problems, if any. HCPs will then perform a physical examination and evaluate the general children’s response and development.

If there are any growth problems discovered during the routine health screening, the child will be referred to a medical officer by the nurse. Subsequently, a referral will be made by the medical officer to other HCPs including a dietitian, nutritionist, paediatrician, or family medicine specialist, if necessary, for further treatment. If a growth problem is detected by the medical officer, the child will be referred directly to the respective HCPs for further action.

In addition, home visits (if required) and counselling for the family of the affected child will be conducted to explore the home environment as well as teach the caregivers proper feeding techniques for the child. The next appointment is then given to the caregivers at a closer date for more frequent growth monitoring of the affected child.

**Fig. 1:**
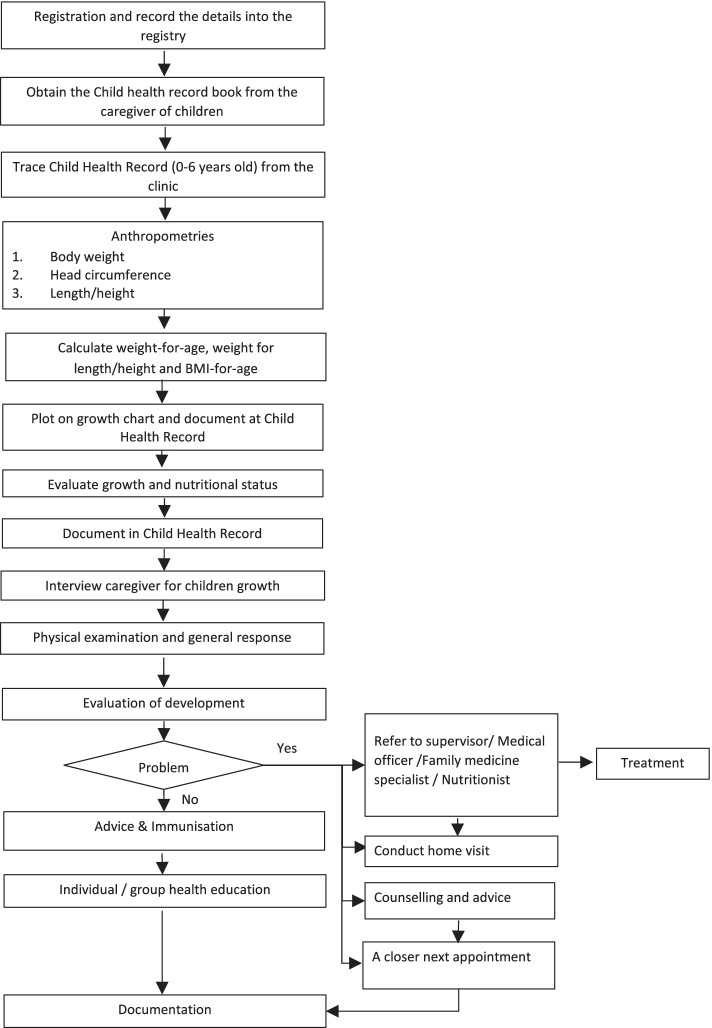
A summary of the standard care flow chart for assessing child growth and development in government-funded health clinics

If there is no growth problem, the child will receive immunization appropriate for age. All mothers will be given health education on children’s growth (Fig. 1) [20, 24].

### Audit standard and criteria

A meeting with stakeholders, which included the state Deputy Director of Public Health, Senior Paediatrician, State Head Matron, Medical Officers, and Nutritionist, was conducted to obtain a consensus in regard to the audit criteria and standards before the commencement of the audit. The audit criteria were set based on current growth management guidelines that all growth charts must be correctly and completely plotted. Children detected as underweight should be managed with appropriate growth management [24]. For this audit, we decided to audit only the growth status of the children; the children’s developmental status was not audited.

Given the high prevalence of underweight *Orang Asli* children [25], and the aim of ‘weight-for-age’ growth charts is to assess underweight or severely underweight young children [19], the stakeholders agreed to only audit ‘weight-for-age’ growth charts of all *Orang Asli* children less than 2 years of age for correctness and completeness of growth chart plotting, and the appropriateness of growth management for *Orang Asli* children who were underweight.

### Instruments and training for data collections

An audit form was created to collect information about the children’s age and to assess growth chart plotting and appropriateness of growth management of children with underweight problem. A total of 29 senior nurses who have been working at the mother-and-child health unit in the primary care health clinics located in Perak State were identified as auditors. They were invited for a training session to learn the auditing procedure. Simulated ‘Clinic Child Health Record’ and growth charts were used for training. In addition, an audit instruction guide was created to instruct auditors on the audit method and audit form completion.

### Sampling Method & Subject Population

To minimize measurement bias, auditors were assigned to conduct cross-district auditing; as such they were not auditing the clinics they worked at. A formal letter to inform the health district officers about this audit was issued a week before the audit began. On the day of the audit, after excluding the growth charts of children with intellectual impairment, developmental delay, children born prematurely (less than 37 weeks), and children with other disabilities (cerebral palsy, dysmorphism, spina bifida) and chronic diseases (e.g., thalassemia, congenital heart disease), ‘weight-for-age’ growth charts of *Orang Asli* children aged 24 months or less and their ‘Clinic Child Health Record’ were all sampled for the audit. All growth charts obtained were compared with a registry of *Orang Asli* children to ensure that the number of children audited tallied.

### Method of data collection

The number of children’s visits to the clinic for growth monitoring that were documented in the ‘Clinic Child Health Record’ was compared against the registry to ensure that the measurements in the records tallied with the number of visits. The correctness and completeness of the ‘weight-for-age’ growth charts plotted were cross-checked with the anthropometry measurements stated in the ‘Clinic Child Health Record’ at the clinic. Operational definitions were as follows: -

- ‘Correctness’ of plotting was defined as the weight measurements in kilograms (kg), up to one decimal place, plotted accurately for the age and on the right chart (male or female growth chart respectively).
- ‘Completeness’ of plotting a chart was considered when all weight measurements were plotted on the growth chart without any missing points. For instance, ten measurements corresponded to ten points plotted on the growth chart. A missing point is defined as a measurement that was performed and documented in the ‘Child Health Record’ but was not plotted on the growth chart. Any missing point detected will be considered a growth chart that was ‘incompletely plotted’.
- ‘Completely not plotted’ is defined as the situation where all the measurements were not plotted on the growth chart.

**Fig. 2:**
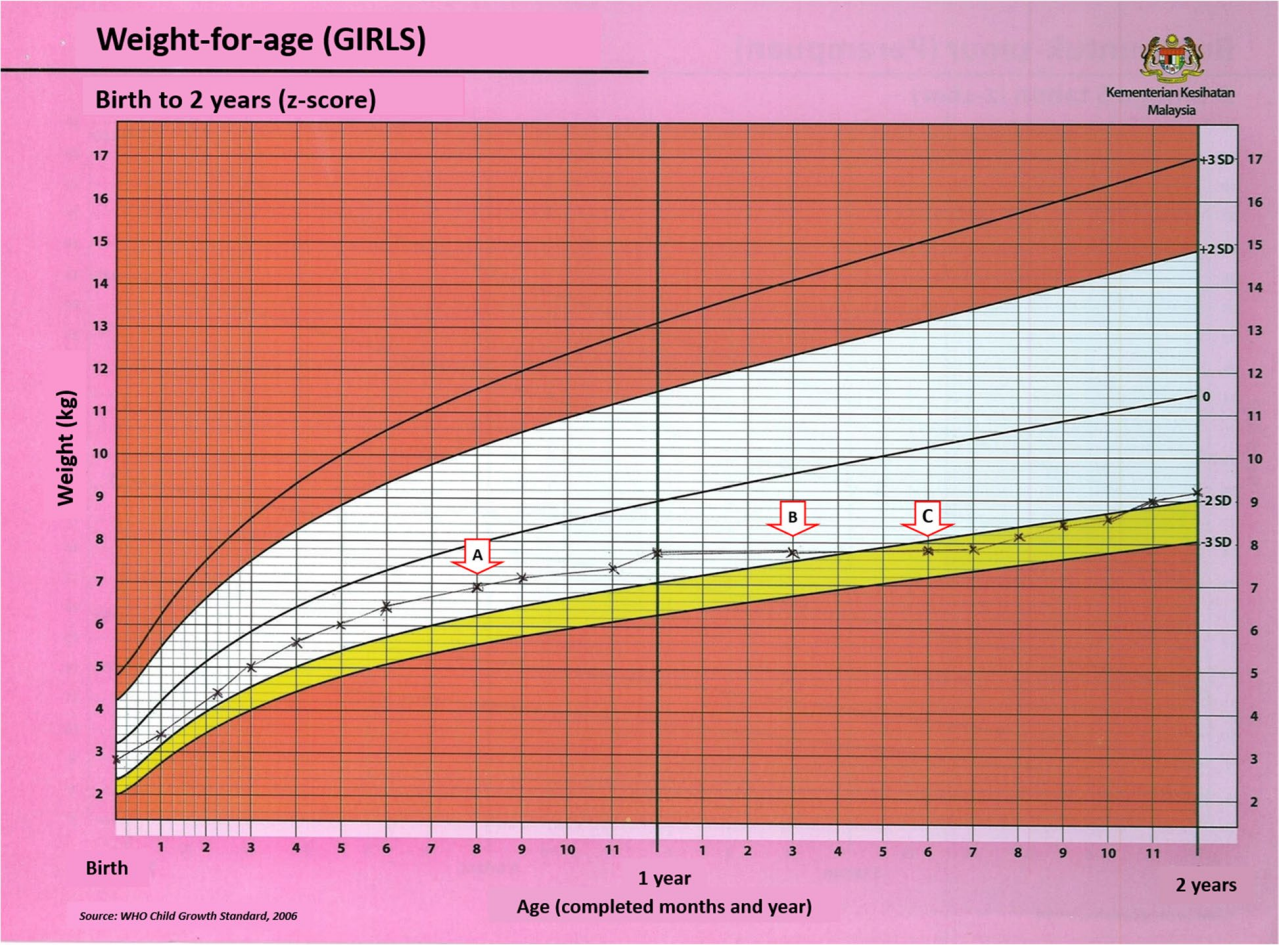
Simulation of ‘weight-for-age’ growth chart of a girl with underweight problem for training purpose. Point A indicates normal growth, point B indicates flat growth line even it is within ±2 z-score, and point C indicates crossing a − 2 z-score line

**Fig. 3:**
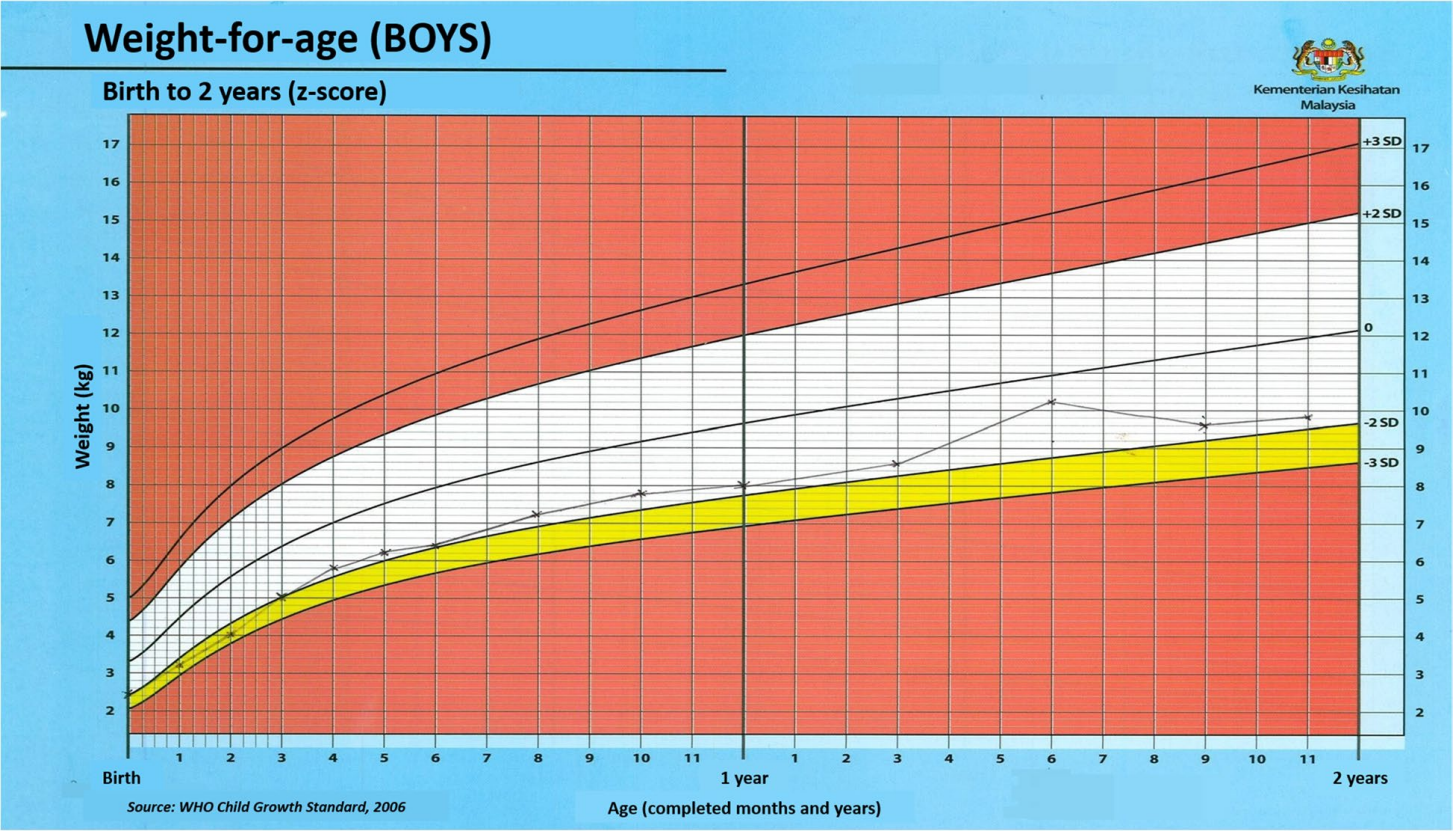
Simulation of ‘weight-for-age’ growth chart of a boy with underweight problem. Point D indicates sharp decline

The growth charts were examined for normal growth trend or underweight problem (crossing a − 2 z-score line, sharp incline or decline in the growth line, or flat growth line). Simulations of “weight-for-age” of a girl (Fig. 2) and a boy (Fig. 3) with normal growth and underweight problems were prepared for the auditors during the training session so as to standardize auditing for data collection. In the following situations, a decision was made about the growth chart according to the data available: -

i. Missing of one point or incorrectly plotted but a measurement of anthropometry was documented in ‘Clinic Child Health Record’, the auditors were allowed to correct and complete the growth chart. The auditors, therefore, could determine the growth status of the child.
ii. Completely not plotted or has many missing points or incorrectly plotted that disallow the determination of growth status, the auditors would state those cases as ‘uncertain status’.

In the case where a child was identified as having an underweight problem, the child’s health record was checked for appropriateness of growth management. Appropriate growth management is defined as HCPs provide suitable managements according to the children’s condition. The type of management included HCPs giving caregivers an earlier follow-up appointment dates (2 to 4 weeks), making referrals to the related health care providers (medical officer, nutritionist, paediatrician, or family medicine specialist), making a home visit to the family of the affected child, and/or providing them with health education. The types of management for underweight included more than one of these interventions above, depending on the child condition. If a child is detected with an underweight problem without any of these appropriate management, the case would be considered inappropriate management. The auditor would inform the health clinic manager to conduct a follow-up action for the child. The completed audit forms were then returned for data analysis.

### Data analysis

The data collected was entered into the Statistical Package for Social Sciences version 20.0. The achievements of audit standards were determined by performing descriptive analysis on the number of growth charts that were not plotted, incompletely or incorrectly plotted, children with normal growth, underweight, and those underweight cases managed with inappropriate growth management in all circumstances. Audit standards were considered not met if there were any episodes of incorrect or incompletely plotting of growth charts or inappropriate management of children who were underweight. The prevalence of underweight children was analysed by identifying the proportion of growth charts with underweight problems.

## Results

A total of 1329 children were included in the analysis after excluding 2 children who were more than 24 months of age. The average age of children was 13.6 months (SD: 5.83) and they were distributed across 32 clinics in 7 districts of Perak State, Malaysia.

### Audit findings

Of 1329 *Orang Asli* children, 527 growth charts were incorrectly or incompletely plotted, and five growth charts were completely not plotted. These growth charts accounted for 40.0% (*n* = 532) of the total growth charts audited. Among these growth charts, 267 (50.2%) children’s growth status was identified as underweight by the auditors, but 265 (49.8%) children’s growth status was beyond the determination of the auditor and determined as ‘uncertain status’. Of 267 underweight children, 199 (74.5%) were identified as having inappropriate growth management. The children categorised as having an uncertain growth status were not able to determine the appropriateness of growth management.

The growth charts that were correctly and completely plotted accounted for 797 (60.0%) children. Of those, 514 (64.5%) were categorised as normal growth, while 283 (35.5%) were found underweight. Of those who were underweight, 194 (68.6%) children were considered to have received inappropriate growth management.

**Fig. 4:**
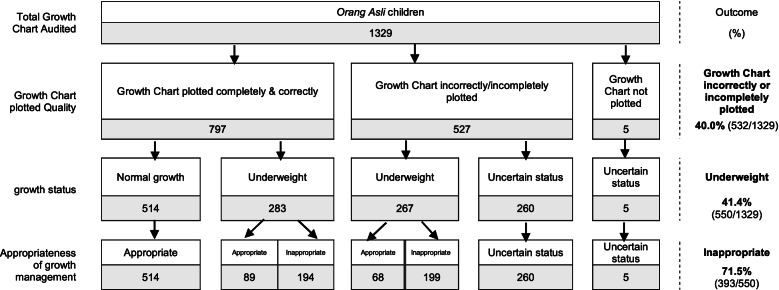
Summary of audit findings

Overall, the total number of underweight *Orang Asli* was 550 (41.4%), of which 393 (71.5%) of them were provided with inappropriate growth management. The summary of audit findings for the growth chart plotting quality, growth status, and appropriate growth management is shown in Fig. 4.

Of 393 (71.5%) underweight children with inappropriate management, 55.5% were not referred to the medical officer, 42.0% did not have a home visit, and 38.7% did not have the next clinic appointment scheduled timely (Table 1).

**Table 1:** Types of inappropriate growth management

Variables	n (%), (*n* = 393)
Types of inappropriate management	
Staff nurses	
No timely clinic appointment	152 (38.7)
No referral to medical officer	218 (55.5)
No home visit	165 (42.0)
No health education given	105 (26.7)
Medical officers	
No referral to nutritionist	73 (18.6)
No referral to paediatric department	34 (8.7)

The audit results showed that less than two-thirds (60%) of the growth charts were correctly plotted and only 28.5% of the underweight children were managed with appropriate growth management. These percentages were below the standard of 100%, which means the HCPs did not achieve the standard of care (Table 2).

**Table 2:** The achievements of audit standards

Audit criteria	Total number	Number of cases met the standard	Achievement	Standard	Outcome
1. Growth chart correctly and completely plotted	1329	797	60.0%	100%	Standard not achieved
2. *Orang Asli* children with underweight received appropriate growth management	550	157	28.5%	100%	Standard not achieved

## Discussion

The evaluation of growth management of *Orang Asli* children who live in Peninsular Malaysia is important in view of the fact that malnutrition, especially underweight children, is a common problem in the *Orang Asli* population. This problem persists, as seen in a few recent reported studies [25–28], despite various measures taken by the Ministry of Health Malaysia [29–31]. This study provides an insight into the extent of inadequacy in growth management of *Orang Asli* children, and could be replicated in other regions [14]. In general, growth management of indigenous children should be evaluated on a regular basis to identify shortcomings, if any, so that prompt intervention can be taken in time to prevent morbidity and mortality among these children.

This study showed that at least 40% of *Orang Asli* children were underweight by the age of 2 years. Various initiatives, including special health care services, such as the *Orang Asli* Mobile Clinic and community feeding for the *Orang Asli* children, have been specifically introduced by the Ministry of Health Malaysia to provide health service and nutrition access to the *Orang Asli* communities [30, 32]. Nevertheless, the high prevalence of underweight in these children remains. Similar to the findings of other studies, the prevalence of underweight problems among *Orang Asli* children (32.7 to 65%) [7–12] was higher than that of children in the general population (15.3%) [25]. The problem is primarily one of the poor socio-economic status of the *Orang Asli* populations when compared to the general population, with high hard core poverty rates [33]. The vicious cycle of underweight problems recurs when the children who have been treated in the hospital are sent back to their underprivileged environments, where their families cannot afford to buy nutritious food for them [34].

Almost 40% of the growth charts were incorrectly and incompletely plotted. This finding was a major concern as the growth chart is the key step for interpreting the growth status [19]. An incorrect or incomplete plot on the growth chart would result in an inaccurate interpretation of growth status and could cause delayed intervention. This study was not planned to look at reasons for poor documentation of growth. Reasons for this need to be identified and remedied.

The audit findings showed that more than two-thirds of underweight children were associated with inappropriate growth management. The factors relating to the inability of HCPs to manage the growth of *Orang Asli* may be multifactorial; this remains to be investigated. The postulated reason for these findings could be explained via each type of growth management as follows: -

(i) Among the underweight children with inappropriate management, not making a referral to the medical officer accounted for the highest rate of inappropriate growth management. There is a lack of clear instruction for HCPs about when a referral should be made for the growth of *Orang Asli* children [20, 24], who are more prone to underweight problems than other ethnic populations in this country [9]. Health policymakers should consider revising the guideline by including a more intensive intervention, including allowing referral to be made when the growth of *Orang Asli* children is suggested to be at risk of inadequate growth, even if the plot is within normal growth. This recommendation should be considered for use in any indigenous community, as evidenced by the indigenous population with significantly higher rates of infant and child mortality, perinatal mortality, low birth weight, and age-standardized death rates [35].
(ii) More than one-third of the underweight cases in this audit were not given timely appointments to attend the clinic. This may be associated with the difficulties of the *Orang Asli* community in seeking healthcare on a regular basis. Cultural and social determinants of health influence indigenous communities’ access to health care [35, 36]. The majority of *Orang Asli* live in poverty [7, 33, 37], may be resulting in their lacking the financial capability to pay for transportation or accommodation fees needed for accessing healthcare. Also, the HCPs could consider proactive identify all *Orang Asli* children with severe malnutrition and admit them to refeeding centers [6, 38].
(iii) A lack of home visits in about 40% of cases could be partly associated with the semi-nomadic lifestyles among some of the tribal groups of *Orang Asli* population and others living in relatively remote areas [39], Geographical logistics limitations could be a factor that causes difficulties for HCPs in locating the family of the affected child and resulting in inadequacy of home visits.
(iv) One-fifth of the cases were not given health education. One of the main cultural elements for a given community is language. Different tribes of *Orang Asli* have their own language system [39], suggesting that a potential language barrier could be the existence between indigenous communities and non-indigenous HCPs [35]. Although the Ministry of Health Malaysia has established special allocating health services for the *Orang Asli* community, such as a mobile clinic for *Orang Asli* [40], ineffective communication due to language barriers may result in undesired healthcare outcomes.

The overall audit achievements were below the set standard, with less than one-third of the cases being provided with an optimal growth management pathway. Suggestions to improve growth management knowledge should consider referring to the practices in other countries, such as Australia. One strategy includes the presence of cultural training for HCPs providing services indigenous people, which is lacking locally [35, 41]. The potential difficulties encountered by HCPs in implementing the growth management guidance could be attributed to the fact that the majority of HCPs were non-indigenous and lacked training in providing health services to communities who practice a distinctively different culture, live in remote, rural geographic settings, and face socioeconomic disparities, such as indigenous people [9, 35, 39, 42, 43]. At the same time, the local method for improving growth management could be adapted from other states in Malaysia, such as Pahang State, which recorded the highest number of *Orang Asli* populations and shows convincing results in improving underweight cases among indigenous children. One of the integrated strategies was the introduction of an *Orang Asli* volunteer training programme in which *Orang Asli* people act as mediators via partnership with the local community nurses or health care providers to help to identify symptoms of health-related problems among pregnant mothers and children who are sick [32]. Involvement of the indigenous community as healthcare providers is not a new idea. It was a successful intervention when *Orang Asli* traditional healers were first recruited in training courses in 1957 to perform simple medical tasks such as dressing and acting as intermediary people to connect medical practitioners to their own community [44]. Given these successful examples, this approach could be adapted to overcome the problem of *Orang Asli* populations being isolated from modern health care.

## Limitations

This audit included all *Orang Asli* children less than 2 years of age who have been followed-up under the growth monitoring program. However, it is possible that the current system is unable to include some of the *Orang Asli* children who are geographically isolated from the known *Orang Asli* villages or communities. Additionally, the exact number of children with special needs excluded from the study was not collected.

The growth management for children from the general population was not evaluated for comparison. It is unclear whether the inadequate growth management was caused by other social-cultural factors such as language barriers, cultural acceptance of the types of growth management and food provided, semi-nomadic lifestyle practices, or religious practices. The real lived experience of health workers in managing the growth of *Orang Asli* children needs further exploration in order to determine their challenges in healthcare delivery to the *Orang Asli* community so that proper measures can be taken to better address the feasibilities in providing some of the care specified in the guidelines. Meanwhile, the barriers preventing accessibility of *Orang Asli* population to primary health care and methods to facilitate them for receiving health care services should be investigated.

## Conclusion

This study showed that the overall growth management was unsatisfactory. The current interventions for managing underweight *Orang Asli* children need to be reviewed to better meet the needs of the *Orang Asli* community. The prevalence of underweight accounted for at least 40% of *Orang Asli* children less than 2 years of age. A more intensive, stepwise guide and strategy for managing underweight *Orang Asli* children should be considered by the stakeholders to improve this problem.

## Data Availability

The datasets generated and/or analysed during the current study are not publicly available due to data confidentiality, but are available from the corresponding author on reasonable request.

## Abbreviations

HCPs: Healthcare providers
